# Interventions to Address Health-Related Social Needs Among People with Kidney Failure: A Rapid Scoping Review

**DOI:** 10.3390/ijerph22091330

**Published:** 2025-08-26

**Authors:** Kathryn S. Taylor, Didi Petkiewicz, Yordanos Tesfai, Deidra C. Crews, Hae-Ra Han

**Affiliations:** 1 Johns Hopkins School of Nursing, Johns Hopkins University, 525 N. Wolfe St., Baltimore, MD 21205, USA; dpetkie1@jh.edu (D.P.); hhan3@jhu.edu (H.-R.H.); 2 Johns Hopkins Bloomberg School of Public Health, 615 N. Wolfe St., Baltimore, MD 21205, USA; ytesfai1@jh.edu; 3 Division of Nephrology, Department of Medicine, Johns Hopkins University School of Medicine, 733 N. Broadway, Baltimore, MD 21205, USA; dcrews1@jhmi.edu; 4 Johns Hopkins O’Brien Center to Advance Kidney Health Equity, Johns Hopkins University School of Medicine, 733 N. Broadway, Baltimore, MD 21205, USA

**Keywords:** social determinants of health, health equity, chronic kidney disease

## Abstract

Background: Globally, socioeconomic disparities persist across the trajectory of chronic kidney disease and are pronounced among people with kidney failure. Unmet health-related social needs contribute to these disparities, but limited guidance exists about how best to address them. To guide implementation, we conducted a rapid scoping review to identify and characterize interventions that address health-related social needs among people with kidney failure. Methods: We adapted established scoping review methods to conduct a rapid review. We searched Embase, PubMed, CINAHL, SCOPUS, and PsychInfo for articles and conference abstracts published since 2013 that described interventions to address health-related social needs as identified in the Centers for Medicare and Medicaid Services’ Accountable Health Communities Health-Related Social Needs Screening Tool. We applied the RE-AIM framework (Reach, Effectiveness, Adoption, Implementation, Maintenance) to synthesize findings and characterize intervention components. Results: Our review identified three articles and five conference abstracts that described diverse interventions to address health-related social needs among people with kidney failure. Six targeted social support, one addressed food insecurity, and one addressed transportation needs. Two pilot studies to address social support reported high recruitment and retention rates. One study formally tested an intervention to address social support among adolescents with kidney failure and reported negative findings (no change in social exclusion). The level of detail about intervention implementation varied across studies, but none described excluded participants or intervention fidelity, adaptations, or cost. Conclusions: Despite recent attention, there remains a lack of evidence to guide interventions addressing health-related social needs among people with kidney failure. From limited available data, interventions to address social support may be feasible and acceptable.

## 1. Introduction

Chronic kidney disease is a major public health concern with a global prevalence of approximately 10% [1]. End-stage kidney disease, or kidney failure, is increasing worldwide due to epidemiological transitions (e.g., population aging and increases in noncommunicable diseases such as hypertension, diabetes and obesity) and expanded availability of renal replacement therapies [2]. From 1990 to 2017, the number of people with kidney failure initiating dialysis or receiving a kidney transplant increased by 43.1% and 34.4%, respectively [3]; and average global prevalence of treated kidney failure is now 823 per million population [4]. Within and across countries, socioeconomic disparities persist across the trajectory of chronic kidney disease and are pronounced among people with treated kidney failure [4,5,6]. In the United States, for example, neighborhood poverty is associated with worse dialysis survival [7], quality of dialysis care [8], and kidney transplant waitlisting and outcomes [9]. Due to structural factors such as structural racism, a higher proportion of Black or Hispanic individuals with kidney failure live in disinvested and high-poverty neighborhoods compared to White individuals (62.2% or 65.4% versus 26.6%) [10].

People with kidney failure, particularly those from historically disadvantaged groups, often experience unmet health-related social needs [11]. The United States Centers for Medicare and Medicaid Services (CMS) define health-related social needs as “an individual’s unmet, adverse social conditions that contribute to poor outcomes” [12]. Health-related social needs include food insecurity, housing instability, community safety concerns, difficulty paying utilities, and challenges with transportation. Recent estimates of food insecurity among people receiving hemodialysis in the United States range from 35 to 60 percent [13,14]. A body of research is beginning to demonstrate the impact of health-related social needs on dialysis outcomes such as survival, hospitalization, and health-related quality of life [15,16,17]. At the same time, major reports such as the National Academies of Sciences, Engineering, and Medicine’s *Integrating Social Care into the Delivery of Health Care: Moving Upstream to Improve the Nation’s Health* have called for healthcare systems to address patients’ unmet social needs [18]. Screening for health-related social needs is now a health system strategy to reduce acute care spending and advance health equity among patient populations with chronic disease, and CMS requires dialysis facilities to conduct such screenings as part of value-based care [19].

Despite recent attention, limited guidance exists about approaches to address health-related social needs among people with kidney failure [20]. A recent systematic review documented that peer support interventions may improve patient-level outcomes like self-efficacy and psychological well-being for this population [21]. However, such interventions have not directly targeted upstream health-related social needs. Moreover, to advance health equity, an understanding of how interventions to address health-related social needs are delivered is needed, in addition to what these interventions entail [22]. Frameworks from implementation science, defined as “the scientific study of methods to promote the systematic uptake of research findings and evidence-based practices into routine practice” [23], can guide this discovery. Given this context, we conducted a rapid scoping review to identify interventions that address health-related social needs among people with kidney failure. We applied the RE-AIM framework (Reach, Effectiveness, Adoption, Implementation, Maintenance) [24], an established implementation science framework, to synthesize findings and characterize intervention components.

## 2. Methods

### 2.1. Design

While scoping reviews and systematic reviews both synthesize existing research on a particular topic, they differ in that the main purpose of scoping reviews is to identify knowledge gaps rather than to answer a specific research question [25]. Further, rapid reviews are “a form of knowledge synthesis that accelerates the process of conducting a traditional systematic review through streamlining or omitting a variety of methods to produce evidence in a resource-efficient manner” [26]. We followed established scoping review methodology from the Joanna Briggs Institute [27]. Given that payers in the United States are currently requiring dialysis facilities to screen patients for health-related social needs as part of value-based care, we adapted our approach to article screening for timeliness as described below. We report our search and results in alignment with the Preferred Reporting Items for Systematic Reviews and Meta-Analyses for Scoping Reviews (PRISMA-ScR) checklist [28].

### 2.2. Search and Selection

In collaboration with an informaticist, we developed a search strategy to identify recent relevant scientific and grey literature, including conference abstracts. We identified health-related social needs from the CMS Accountable Health Communities Health-Related Social Needs Screening Tool, including food insecurity, housing instability, transportation challenges, difficulty paying utilities, financial strain, and social support [29]. In December 2023, we conducted a search of Embase, PubMed, CINAHL, SCOPUS, and PsychInfo for articles and conference abstracts written in English and published since 2013 (see Supplemental Table S1 for the full search terms and yields from each database search). We applied the PICOS (population, intervention, comparison, outcome, study type) framework to define study eligibility criteria [30], and included articles or conference abstracts of intervention studies if they addressed health-related social needs among people with kidney failure (Table 1). Conceptually, the Health-Related Social Needs Screening Tool screens for social support needs with one item related to instrumental social support (“…If for any reason you need help with day-to-day activities…do you get the help you need?”) and one related to loneliness or social isolation (“how often do you feel lonely or isolated from those around you?). Therefore, we conceptualized interventions to promote social connection or engagement as “interventions to address social support.” We included studies regardless of which type of kidney replacement therapy participants were receiving, and regardless of outcome. We included randomized and nonrandomized studies. We excluded studies of people with earlier stages of chronic kidney disease.

**Figure 1:**
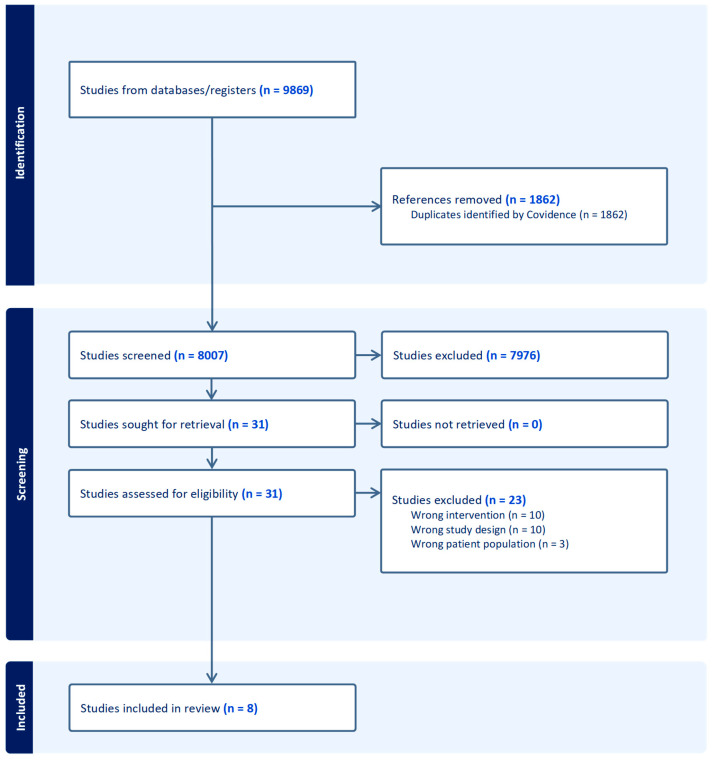
PRISMA Diagram.

Our literature search resulted in 9869 studies. After automated removal of duplicates, we screened titles and abstracts for 8007 unique studies. A team of three trained reviewers each independently screened approximately 600 titles and abstracts and met biweekly to discuss findings and establish consensus. Subsequently, one reviewer screened remaining titles and abstracts. Fully, 31 articles or conference abstracts were selected for full-text screening. At this phase, we screened reference lists from selected articles but did not identify other potentially eligible studies. Two reviewers (DP and KT) independently screened each full-text article or conference abstract for inclusion and resolved discrepancies via discussion. Twenty-three articles were excluded: three studies were excluded due to their population, ten studies due to their intervention, and ten studies due to their design. Finally, eight studies met inclusion criteria and were included in the scoping review (Figure 1).

### 2.3. Data Extraction and Synthesis

We applied the RE-AIM framework to guide data extraction and synthesis. Since it was constructed in 1999, the RE-AIM framework has expanded to incorporate contextual factors that impact RE-AIM domains and to explicitly address health equity (Supplemental Figure S1) [24]. Each of the five RE-AIM domains includes assessment questions for intervention planning or evaluation. We adapted a data extraction form from a scoping review by Hirchak and colleagues that used RE-AIM assessment questions to synthesize included studies and incorporated a health equity lens [31]. For example, one assessment question related to Reach was, “Authors describe engagement strategies or equity issues related to participation.” Two reviewers (DP and KT) piloted and refined the extraction tool before use via discussion and consensus.

## 3. Results

### 3.1. Description of Studies

Of the eight studies included in this review, three were full-text articles [32,33,34] and five were conference abstracts (Table 2) [35,36,37,38,39]. Seven were conducted in the United States [33,34,35,36,37,38]. Of those, six were conducted in cities with large academic medical centers [33,34,35,36,38]. Of the three full-text studies reporting funding, one was funded by the Dutch Kidney Foundation [34], one by the National Institute on Aging [33], and one by a university [32].

Two of the studies in the review were pilot studies (a single-group prospective study [34] and one randomized controlled trial [33]) and six were program evaluations or quality improvement projects [32,35,36,37,38,39]. Only the randomized controlled trial enrolled intervention and control groups [33]. Of the seven studies with single groups, three studies [32,36,37] used a pre-post design and the remaining four studies reported post-intervention outcomes [35,37,38,39]. Except for one program evaluation with 2964 patients [35], studies had small sample sizes ranging from 10 to 34 participants. Four studies purposefully sampled socially disadvantaged groups with kidney failure. Of those four, samples included undocumented immigrants [34], older adults with low socioeconomic status [33], adults obtaining food bank bags from the dialysis facility [38], and children living in minoritized communities [36]. The remaining studies did not provide details about participant socioeconomic status.

Intervention characteristics and study outcomes varied across studies. Six out of eight studies targeted social support [32,33,34,36,37,39]. One program targeted food insecurity [38] and a separate program targeted transportation needs within a broader care coordination service [35]. Of the six studies targeting social support, three delivered the intervention via participant peers [32,34,37]. In two studies, participant peers were defined based on demographic and social characteristics (e.g., adolescents, undocumented immigrants) [32,34]. The remaining three studies delivered the intervention via medical students [39] or multidisciplinary teams. In the studies involving multidisciplinary teams, one delivered the intervention via a triad of nurse, occupational therapist, and handyman [33]. The other was a unit-based “Psychosocial Power Team,” including nursing, massage therapy, recreational therapy, social work, and psychology [36]. The two studies addressing food insecurity or transportation needs featured organizational partnerships (e.g., a dialysis facility and a food bank) [35,38]. Participants received interventions at healthcare facilities (dialysis facility or hospital) [34,36,37,38,39], their own homes [33], or a remote camp [32]. When reported in detail, intervention duration lasted between one week [32] and six months [34]. Both pilot studies and two program evaluations reported some aspect of intervention feasibility and acceptability [33,34,38,39]. Four studies evaluated resolution of the health-related social need [32,33,36,38]. Two studies used a validated measure of a health-related social need [32,33]. In the two other studies in which authors measured the targeted health-related social need, they either developed their own measure or did not describe it in detail [36,38]. Two reported clinical outcomes (interdialytic weight gain and visit adherence) [35,37].

### 3.2. Application of RE-AIM Framework

Table 3 presents RE-AIM data extracted from the three full-text articles included in this review. We were unable to extract RE-AIM data from conference abstracts because reporting was inherently limited due to word count restrictions. Each of the three full-text articles used multi-method or mixed methods designs (i.e., quantitative and qualitative methods) to evaluate reach, effectiveness, adoption, implementation, or maintenance. Regarding reach, none of the articles described individuals who were excluded from studies or interventions. One study detailed recruitment via a culturally concordant study team member [34]. Regarding effectiveness, none of the studies measured attrition by participant characteristics; however, two studies targeted structurally disadvantaged groups (undocumented immigrants and Black older adults with low incomes) and reported reasons for participants leaving the studies [33,34]. Regarding adoption, all three studies discussed the number of staff who delivered the interventions, but only one study described the representativeness of the staff member [34]. None of the studies provided details on intervention fidelity, adaptations, or cost. One study discussed a plan for intervention sustainability via establishment of a non-profit organization [34].

**Table 3:** RE-AIM Domains Characterized in Full-Text Studies.

	Cervantes (2023) [34]	Crews (2019) [33]	Sattoe (2013) [32]
**Reach**			
*Authors reported characteristics of excluded individuals*	No	No	No
Authors described engagement strategies or equity issues related to participation	Yes: Culturally concordant study team member conducted in-person recruitment	No	No
Authors use qualitative methods to understand reach or recruitment	No	No	Yes: Semi-structured interview elicited rationale of referring nephrologists
**Effectiveness**			
Authors identify primary outcome	Yes: Feasibility and acceptability	Yes: Feasibility and acceptability	Yes: Self-efficacy, self-management; autonomy in social participation
Authors discussed effectiveness across subgroups	n/a: Study targeted a subgroup	n/a: Study targeted a subgroup	No
Authors measured broader outcome (e.g., quality of life)	No	No	Yes: Health-related quality of life
Authors measured short-term attrition by participant characteristics	n/a: Study targeted subgroup; reported reasons for attrition	n/a: Study targeted subgroup; reported reasons for attrition	No
Authors used qualitative methods to understand outcomes	Yes: Structured interview to elicit value of peer support intervention	Yes: Focus groups to inform adaptation of existing intervention and potential value of adapted version	Yes: Semi-structured interview elicited value of camp and mechanisms of action
**Adoption**			
Authors discussed number and representativeness of staff who delivered the program	Yes: One culturally concordant study staff member led peer support group meetings	Yes: 3 staff per participant; no description of representativeness	Yes: 1-to-1 ratio of “buddy” to attendee; no description of representativeness
Authors reported characteristics of participating and non-participating settings	No	No	No
Authors used qualitative methods to understand staff participation	n/a: Intervention delivered by study staff	No	Yes: Semi-structured interview elicited “buddy” experience
**Implementation**			
Authors reported intervention fidelity	No	No	No
Authors reported adaptations made to intervention during study	No	No	No
Authors reported adaptations that might be needed to promote equity	No	No	No
Authors reported intervention cost (time or money)	No	No	No
Authors reported multi-level context that either facilitated or hindered implementation	Yes: Enrolled participants from hospital on same day of the week; participants preferred hospital setting for group meetings	No	No
Authors used qualitative methods to understand implementation	Yes: Semi-structured interview elicited participants’ perspectives about value of peer support group	No	Yes: Semi-structured interviews elicited “buddy” experience with implementation
**Maintenance**			
Authors reported primary outcome, broader outcome, subgroup effects, or attrition over the long-term	n/a: Pilot study	n/a: Pilot study	No
Authors discussed alignment to organizational mission or sustainability of business model	Yes: Participants formalized peer support group via nonprofit organization and social media	No	No
Authors use qualitative methods to understand setting level institutionalization	Yes: Semi-structured interviews elicited participants’ motivation for starting a nonprofit	No	No

## 4. Discussion

Our scoping review identified a nascent evidence base for interventions addressing health-related social needs among people with kidney failure. In small pilot studies, interventions to address health-related social needs were feasible and acceptable to participants [33,34]. Single group program evaluations targeted different populations across varying levels of socioeconomic status. They intervened upon different health-related social needs, and used different measures for social needs and outcomes. This heterogeneity limits our ability to draw conclusions about intervention effectiveness and generalize findings. Of the five domains in the RE-AIM framework, the three full-text studies provided some detail about effectiveness and adoption, but limited detail about reach, implementation, and maintenance.

We conducted our literature search in December 2023. Since that time, new projects have formed to address socioeconomic disparities in kidney failure and dialysis by addressing health-related social needs and other contributing factors. For example, as part of the Eliminating Racism and Structural in Equities in Kidney Disease (ERASE-KD) Consortium, a multidisciplinary team in New York began testing a community health worker (CHW) intervention to help people with kidney disease address health-related social needs and navigate the kidney transplantation process (https://erasekd.org/ (accessed on 28 January 2025)) [20]. Unfortunately, project funding from the United States National Institutes of Health was abruptly terminated in May 2025 [40]. Additionally, an abstract presented in April 2024 at the World Congress of Nephrology reported results from a randomized controlled trial of a CHW intervention in Colorado with Latinx individuals with kidney failure. Between one-fifth and one-third of participants reported difficulty accessing food, housing, transportation, medicine, or paying for utilities in the past year. The abstract did not report resolution of health-related social needs, but the study did find differences in levels of patient activation between intervention and control groups post-intervention [41]. Lastly, an abstract presented by Novick and colleagues at the American Society of Nephrology Kidney Week in 2024 described a pilot CHW intervention with 17 dually eligible adults receiving dialysis in Texas. Participants had high rates of health-related social needs at baseline (e.g., 70% reported housing instability) though post-intervention measures were not reported [42].

Multisector partnerships and CHW interventions are promising mechanisms to address unmet social needs and advance health equity in the dialysis population. In most studies included in our review, either participant peers [32,34,37] or medical providers (or medical students) [33,36] delivered the interventions, raising concerns about replicability, costs, scalability, and sustainability. In particular, peer-delivered interventions (e.g., for peer support) are impactful but may lack formal structures needed for scale [21]. In the context of health-related social service delivery for people who have traditionally been underserved due to structural inequities such as racism [43], CHWs are quickly emerging as effective agents in addressing their needs. According to a recent return-on-investment analysis, every dollar invested in a CHW intervention addressing social needs would return $2.47 to an average Medicaid payer within the fiscal year [44]. Medicare has begun to reimburse CHW services as detailed in the Calendar Year 2024 Physician Fee Schedule, creating a pathway for scalability and sustainability.

Our review has important implications for future research and practice. In particular, more rigorous study designs that include pre- and post-testing, control groups, and/or randomization are needed, powered by larger sample sizes [20]. When researchers test interventions to address health-related social needs, they can use waitlist control groups to address ethical concerns and use standardized measures. They can apply implementation science frameworks to enhance reporting about implementation, strengthen external validity, capture information about process, and promote sustainability. Researchers should be open to reporting intervention cost, adaptations, and implementation challenges. Additionally, they should capture health-related social needs and clinical outcomes to deepen our understanding of causal pathways. Lastly, research to address health-related social needs should explore the role of dialysis facilities and large dialysis organizations. Beginning in 2025 via the Quality Incentive Program, CMS will hold dialysis facilities accountable for collecting data on food insecurity, housing instability, transportation needs, utility issues, and interpersonal safety [19]. These changes suggest that dialysis facilities may soon be held accountable for addressing health-related social needs within their patient populations, though the evidence base for how to do so is underdeveloped. Regardless of reporting requirements, the kidney community must do the hard work of learning new ways to care for people with kidney failure and complex social needs. A recent report from the National Institutes of Diabetes and Digestive and Kidney Diseases outlined potential solutions, including multi-level interventions (i.e., at patient, provider, and community level), improved data collection on unmet health-related social needs, and integrating community health workers with the healthcare team [45].

This study has some important limitations. A single reviewer conducted the majority of title and abstract screenings and may have inadvertently excluded relevant studies [46]. The reporting of studies included in the conference abstracts was inherently limited in the information provided. These limitations are balanced by key strengths, including the expansiveness and systematic nature of our literature search and application of an implementation science framework to guide synthesis of findings. Moreover, we are not aware of existing reviews of interventions to address health-related social needs among people with kidney failure, particularly among historically disadvantaged groups.

In conclusion, despite recent attention, there remains a lack of evidence to guide interventions addressing health-related social needs among people with kidney failure. New research is urgently needed given the high burden of health-related social needs in this population and the persistence of health inequities. Implementation science frameworks can help ensure that new research is externally valid and that interventions are adopted and sustainable.

## Figures and Tables

**Table 1:** Study Eligibility Criteria.

	Inclusion	Exclusion
Population	People with kidney failure, including those on hemodialysis (home or in-center) or peritoneal dialysis and those who have received a kidney transplant From/living in any country Within any age group	People with earlier stages of chronic kidney disease
Intervention	Interventions that address HRSNs, including food insecurity, housing instability, transportation, utilities, financial strain, and social (family or community) support Can be part of a multi-level or multi-component intervention	Interventions not addressing health-related social needs, for example, patient education or behavioral interventions
Comparison	n/a	n/a
Outcome	Any outcome	n/a
Study Type	Any intervention design involving “treatment” of the HRSN variable, including RCT, quasi-experimental, and pilot studies Can include mixed methods studies Can include conference abstracts	Observational designs where the HRSN is not intervened upon, for example, correlational studies Case studies Non-research articles, for example, editorials, narrative reviews

HRSN: Health-Related Social Need.

**Table 2:** Characteristics of Included Studies.

Author (Year) Setting Funding	Sample	Study Objective HRSN & Measure Study Design	Intervention (I) Control (C)	Select Outcomes
Barrera et al. (2013) [39] * Stanford, CA United States	5 pairs of medical students and outpatient pediatric ICHD patients Sample characteristics not described	To evaluate structured program to address psychosocial factors affecting adherence and morbidity among pediatric ICHD patients Social support; not measured Program evaluation; single group, post-program only	I: Program to pair medical students with chronically ill children to provide social support; met weekly or biweekly in dialysis unit to engage in social activities (e.g., board games)	Abstract reports general, positive description of program experience from patients, parents, students, and nurses
Cervantes et al. (2023) [34] Denver, CO United States Funding: Internal, University of Colorado School of Medicine	Undocumented immigrants with kidney failure who were hospitalized for emergency dialysis n = 23 Age, mean, y 47 61% male 100% Hispanic 61% < high school education	To investigate the feasibility and acceptability of a single-group peer support group intervention Social support; not measured Single group prospective study	I: Six-month, hospital-based peer support program with weekly, biweekly, or monthly 90 min support group meetings	Feasibility: 85.2% recruitment rate 78.3% retention rate Importance of camaraderie and emotional support from peers elicited in structured interviews
Crews et al. (2019) [33] Baltimore, MD United States Funding: National Institute on Aging, National Institute of Health	Older adults with kidney failure, treated with ICHD in Baltimore City, ≥1 limitation in physical functioning, and low socioeconomic status n = 12 I: n = 6 Age, mean, y 69.5 66% male 100% Black 33% < high school education C: n = 6 Age, mean, y 68.6 50% male 100% Black 50% < high school education	To pilot test a home-based program to improve physical and social functioning of low socioeconomic status older adults treated with hemodialysis Social support; Lubben Social Network Scale-Revised Single-blind, two-group, randomized feasibility trial	I: Adaptation of CAPABLE, a home-based program for older adults; five-month program including up to six visits with occupational therapist, up to five visits with nurse, and full day of work from handyman for home repairs C: Usual care, waitlisted	Feasibility: 1 month recruitment timeframe 100% completion rate of those alive at the end of the study Baseline vs. five months post-intervention: Social Network score improved (+4.8, combining intervention and waitlist control groups)
McCall & Abdel-Rahman (2021) [38] * Charlottesville, VA United States	People with kidney failure receiving hemodialysis and consistently receiving food bank bags at dialysis facility n = 34 Age, mean, y 61.3 42.9% male 64.3% Black Individual-level socioeconomic status characteristics not described	To evaluate the impact of a food delivery program on food insecurity among patients receiving dialysis in a high-poverty community Food insecurity; single item measure about skipping meals due to being short on food Program evaluation; single group, post-program only	I: Partnership with local food bank to deliver renal-friendly meals to dialysis facility	10.7% Skipping meals 85.7% Eating more meals on a regular basis 50% Satisfied with program
Sattoe, Jedeloo, & Van Staa (2013) [32] * The Netherlands Funding: Dutch Kidney Foundation	Adolescents with kidney failure participating in “transition camp” n = 32 Age, mean, y 19.1 53.1% male Individual-level socioeconomic status characteristics not described	To explore the effects of peer-to-peer support on self-management among young people with kidney failure participating in “transition camp” Social support; DISABKIDS condition generic questionnaire, social inclusion and social exclusion domains Mixed methods program evaluation; single group, pre-post design	I: One-week peer support camp program for adolescents with kidney failure transitioning to adulthood; “buddies” (adults with kidney failure) engage with “attendees” (adolescents with kidney failure) to improve disease self-management	Camp start vs. camp closure: Social inclusion worsened (–7, *p* < 0.05), no change in social exclusion
Varghese (2021) [37] * Riverside, CA United States	Sample not described	To implement and evaluate the effect of social support on fluid restriction adherence among people with kidney failure receiving ICHD Social support; not measured Quality improvement project; single group, pre-post design	I: “Social support person” to improve fluid restriction adherence; limited description of intervention in abstract	Pre- vs. post-intervention: Mean interdialytic weight gain worsened (+0.18 kg)
Whaley et al. (2022) [36] * Columbus, OH United States	Children with kidney failure receiving ICHD and living in minoritized communities n = 16 Sample characteristics not described	To pilot and evaluate research-based psychosocial interventions to support social development and mental health for children on hemodialysis Social support; percentage of patients requiring psychology follow-up for psychosocial concerns Program evaluation; single group, pre-post design	I: Interdisciplinary “Psychosocial Power Team” created patient-specific and unit-wide treatment goals to support patient coping and adjustment; interventions included milestone celebrations	Pre- vs. post-intervention: 65% fewer patients required psychology follow-up
Zheng et al. (2020) [35] * San Francisco, CA United States	Patients 1 to 3 years post kidney transplant receiving care at a Transplant Nephrology Clinic (TNC, a collaboration between an integrated healthcare system and transplant center) n = 2694 Sample characteristics not described	To evaluate adherence and quality of care among patients at the TNC Transportation, “social services”; not measured Program evaluation; single group, post-intervention only	I: TNC connects patients and Transplant Centers with travel and lodging, provides social services	98% adherence to clinic visits

* Conference abstract. ICHD, In-center hemodialysis; HRSN, Health-Related Social Need.

## Supplementary Materials

The following supporting information can be downloaded at: https://www.mdpi.com/article/10.3390/ijerph22091330/s1, Figure S1: RE-AIM and PRISM Frameworks with Equity Lens; Table S1: PubMed Search Strategy.
