# Supplementary material for: Interventions to Address Health-Related Social Needs Among People with Kidney Failure: A Rapid Scoping Review
Source: Int J Environ Res Public Health. 2025 Aug 26;22(9):1330. doi: 10.3390/ijerph22091330 (PMC12469606; doi:10.3390/ijerph22091330)

Supplemental Table S1. PubMed Search Strategy

Search number	Query	Results
**8**	(#1 AND (#2 OR #3 OR #4 OR #5 OR #6 OR #7)) AND (("2013"[Date - Publication] : "3000"[Date - Publication]))	**2,204**
**7**	"Employment"[Mesh] OR "Unemployment"[Mesh] OR "Employment, Supported"[Mesh] OR "Work"[Mesh] OR employee* [tiab] OR employment [tiab] OR "unemploy*"[tiab] OR "job"[tiab] OR "jobs"[tiab] OR "occupation"[tiab] OR "labour"[tiab] OR "labor"[tiab] OR "salaries"[tiab] OR "salary"[tiab] OR "wage*"[tiab] OR "Labor Force*"[tiab] OR "Precarious Employment"[tiab] OR "Marginal Employment"[tiab] OR "Employment Insecurit*"[tiab] OR "Underemploy*"[tiab]	**476,805**
**6**	"Social Support"[Mesh] OR "Family Support"[Mesh] OR "Community Support"[Mesh] OR "Social Inclusion"[Mesh] OR "family support*"[tiab] OR "community support*"[tiab] OR "social inclusion"[tiab] OR "Social Care"[tiab] OR "community resilience"[tiab] OR "family environment*"[tiab]	**103,963**
**5**	"Household Hardship"[tiab] OR "Utility needs"[tiab] OR " difficulty paying utility bills"[tiab] OR " electric company"[tiab] OR "gas company"[tiab] OR "oil company"[tiab] OR "water company"[tiab] OR "shut off"[tiab] OR "heating off"[tiab:~3]	**2,107**
**4**	"Transportation"[Mesh:NoExp] OR "Transportation of Patients"[Mesh] OR "Patient Transportation"[tiab] OR "Access Transportation"[tiab:~3] OR "Transportation Accessibility" OR "Accessible Transportation"[tiab] OR "afford transportation"[tiab:~3] OR "affordable transport*"[tiab] OR "unaffordable transportation"[tiab:~1] OR "reliable transportation"[tiab:~3] OR "unreliable transport*"[tiab] OR "lack transportation"[tiab:~3] OR "lacking transportation"[tiab] OR "lack vehicle"[tiab:~3] OR "lack license"[tiab:~3] OR "shut in"[tiab] OR "shut ins"[tiab] OR "home bound"[tiab] OR "public transport*"[tiab]	**34,385**
**3**	"Food Insecurity"[Mesh] OR "Food Ration*"[tiab] OR "food securit*" OR "food insecurity*"[tiab] OR "malnourish*"[tiab] OR "undernourish*"[tiab] OR "under nourish*"[tiab] OR "underfed"[tiab] OR "nutrition securit*"[tiab] OR "nutrition insecurit*"[tiab] OR "nutrition inadequa*"[tiab] OR "nutrition adequa*"[tiab] OR "food accessib*"[tiab] OR "food availab*"[tiab] OR "food desert*"[tiab] OR "food scarcity"[tiab] OR "food unsustainability"[tiab:~3] OR "food inadequa*"[tiab]	**49,050**
**2**	"Ill-Housed Persons"[Mesh] OR "Housing Instability"[Mesh] OR "Home Environment"[Mesh] OR "homeless"[tiab] OR "ill-house*"[tiab] OR "unhoused"[tiab] OR "houseless"[tiab] OR "hard to house"[tiab] OR "lack of housing"[tiab] OR vagabond*[tiab] OR vagrant*[tiab] OR indigent[tiab] OR "couch surf*"[tiab] OR "living rough"[tiab:~3] OR "sleeping rough"[tiab:~3] OR "sleep rough"[tiab:~3] OR "no fixed abode" OR "unstable housing"[tiab:~3] OR "instability housing"[tiab:~3] OR "insufficient housing"[tiab:~3] OR "Housing Insecur*"[tiab] OR "ghetto*"[tiab] OR "Eviction"[tiab] OR "unsheltered"[tiab] OR "street child*"[tiab]	**20,489**

**1**	"Kidney Failure, Chronic"[Mesh] OR "end-stage kidney disease"[tiab] OR "hemodialysis"[tiab] OR "kidney transplant"[tiab] OR "kidney failure"[tiab] OR "renal insufficiency"[tiab] OR "hemodialysis"[tiab] OR "haemodialysis"[tiab] OR "hemofiltration"[tiab] OR "haemofiltration"[tiab] OR "hemodiafiltration"[tiab] OR "haemodiafiltration"[tiab] OR "peritoneal dialysis"[tiab] OR "dialysis"[tiab] OR "predialysis"[tiab] OR "pre-dialysis"[tiab] OR "kidney disease*"[tiab] OR "renal disease*"[tiab] OR "kidney failure"[tiab] OR "renal failure"[tiab]	**441,435**

Supplemental Figure S1. RE-AIM and PRISM Frameworks with Equity Lens[44]

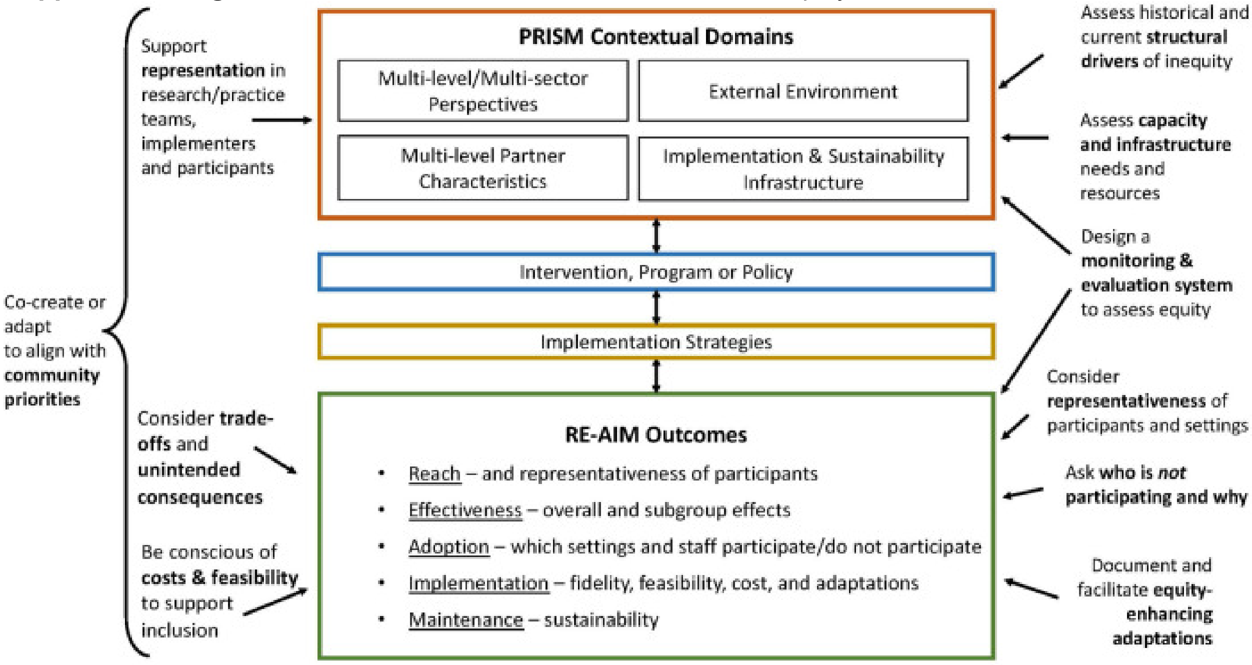

Supplement: Supplementary file 1 [file ijerph-22-01330-s001.zip › ijerph-3763491-supplementary.pdf]
